# HOW IMPORTANT IS IT TO PRAY AND MEDITATE FOR BRAZILIAN OLDER ADULTS? SOCIODEMOGRAPHIC AND CLINICAL CHARACTERISTICS

**DOI:** 10.1093/geroni/igad104.0931

**Published:** 2023-12-21

**Authors:** Mariana Salecker, Yolanda de Souza, Angelo José Bós

**Affiliations:** Pontifical Catholic University of Rio Grande do Sul, Porto Alegre, Rio Grande do Sul, Brazil; Pontifical Catholic University of Rio Grande do Sul, Porto Alegre, Rio Grande do Sul, Brazil; IGG-PUCRS, Porto Alegre, Rio Grande do Sul, Brazil

## Abstract

Praying and meditating are considered spiritual expressions, but little is known about this importance among older adults. Therefore, this abstract aim to understand the frequency and the sociodemographic and clinical characteristics related to the importance given by the Brazilian older adults to praying and meditating. This is a secondary analysis of the transversal data of the Older Adults Health Longitudinal Study, made by Fiocruz in 2015. The dependent variable is: “How much daily praying and meditating is important to you?” Independent variables included: sex, age, educational level, marital status, race, and self-perception of health. The relation between the variables were tested by Chi-Square, considering p<0.05 significant. Praying and meditating everyday were important in 87.6% of the participants. Higher percentages were noticed between the ages of 60 to 69 years old (88.8%) and the ages 80 to 89 years old (88.1%, p<0.001), women (91.6%, p<0.001), widows (90.8%, p<0.001), indigenous (89.1%, p<0.001), with primary school complete (90.5%, p<0.001) and with regular auto perception health (88.4%, p=0.051). We conclude that there is higher than expected frequency of Brazilian older adults who consider important praying and meditating. They are more likely to be women, widow, indigenous, younger, less educated and have regular self-perception of health.

